# Withdrawn: The current state, future supply and demand of therapy physicists ‐ A special report of the 2020 AAPM therapy physicist workforce survey results

**DOI:** 10.1002/acm2.13632

**Published:** 2023-07-03

## Abstract

Withdrawal: Erli Chen, Michael D. Mills, John A. Antolak, Ivan M. Buzurovic, William Dezarn, Farhana R. Khan, Charles Kirby, Brent Parker, Todd Pawlicki, Zhong Su, Chrisine M. Swanson, Russell B. Tarver, Bruce Tomadsen, Nicholai E. Wingreen, Susan White, Ming Yang, Sumin Zhou, “The current state, future supply and demand of therapy physicists ‐ A special report of the 2020 AAPM therapy physicist workforce survey results,” Journal of Applied Clinical Medical Physics, https://doi.org/10.1002/acm2.13632. The above article, published online on 5 December 2022, on Wiley Online Library (wileyonlinelibrary.com), has been withdrawn by agreement between the authors, The American Association of Physicists in Medicine and Wiley Periodicals, LLC. The withdrawal has been agreed due to a technical error at the publisher that caused the article to be mistakenly published online although publication had been canceled because the authors did not approve their proof. The publisher regrets the error. [Correction added on 6 July, 2023 after first online publication: the withdrawal statement has been updated.]

